# Context-Aware Medical Systems within Healthcare Environments: A Systematic Scoping Review to Identify Subdomains and Significant Medical Contexts

**DOI:** 10.3390/ijerph20146399

**Published:** 2023-07-19

**Authors:** Michael Zon, Guha Ganesh, M. Jamal Deen, Qiyin Fang

**Affiliations:** 1 Michael DeGroote School of Medicine, McMaster University, Hamilton, ON L8S 4L8, Canada; 2 School of Biomedical Engineering, McMaster University, Hamilton, ON L8S 4L8, Canada; ganesg1@mcmaster.ca; 3 Department of Electrical and Computer Engineering, McMaster University, Hamilton, ON L8S 4L8, Canada; jamal@mcmaster.ca; 4 Department of Engineering Physics, McMaster University, Hamilton, ON L8S 4L8, Canada

**Keywords:** context-awareness, contexts, past medical history, sensing, telemedicine

## Abstract

Context awareness is a field in pervasive computing, which has begun to impact medical systems via an increasing number of healthcare applications that are starting to use context awareness. The present work seeks to determine which contexts are important for medical applications and which domains of context-aware applications exist in healthcare. A systematic scoping review of context-aware medical systems currently used by patients or healthcare providers (inclusion criteria) was conducted between April 2021 and June 2023. A search strategy was designed and applied to Pub Med, EBSCO, IEEE Explore, Wiley, Science Direct, Springer Link, and ACM, articles from the databases were then filtered based on their abstract, and relevant articles were screened using a questionnaire applied to their full texts prior to data extraction. Applications were grouped into context-aware healthcare application domains based on past reviews and screening results. A total of 25 articles passed all screening levels and underwent data extraction. The most common contexts used were user location (8 out of 25 studies), demographic information (6 out of 25 studies), movement status/activity level (7 out of 25 studies), time of day (5 out of 25 studies), phone usage patterns (5 out of 25 studies), lab/vitals (7 out of 25 studies), and patient history data (8 out of 23 studies). Through a systematic review process, the current study determined the key contexts within context-aware healthcare applications that have reached healthcare providers and patients. The present work has illuminated many of the early successful context-aware healthcare applications. Additionally, the primary contexts leveraged by these systems have been identified, allowing future systems to focus on prioritizing the integration of these key contexts.

## 1. Introduction

Context-aware computing is the notion of using situational and environmental information about users, places, and objects to adapt a computer application to fit a user’s needs [1]. The debate surrounding the precise definition of context awareness dates back to the term’s first use by Schilit and Theimer in 1994 [2]. However, the definitions of context and context awareness that are widely accepted by researchers in the domain of computer science were formulated by Abowd and Dey 5 years later [1]. Regarding context, Abowd and Dey defined it as,

“any information that can be used to characterise the situation of an entity. An entity is a person, place, or object that is considered relevant to the interaction between a user and an application, including the user and applications themselves.”

[1]

With respect to understanding context, Abowd and Mynatt identified the five W’s, namely: who, what, where, when, and why, as the minimum information necessary to determine context [3]. Following the successful definition of context, the application of contexts to computing systems was defined by Dey, who stated,

“A system is context-aware if it uses context to provide relevant information and/or services to the user, where relevance depends on the user’s task.”

[1]

Within a medical setting, a telemedical system is context aware so long as it uses context to change its behaviour in a useful manner. This notion is pivotal in many remote monitoring medical applications, as medical data cannot often be differentiated between being benign and dangerous without context. For instance, a heart rate of 160 could either be caused by sinus tachycardia as a user’s context is exercising or a dangerous arrhythmia given that the user’s context is that they are in bed sleeping. Advanced telemedical systems will need to be developed that not only monitor users’ medical data but also interpret it in a meaningful way. This requirement has led medical researchers to explore how common frameworks within the domain of pervasive computing, specifically context-aware computing, can be utilized by telemedical systems to determine the user context from sensor data to make decisions within medical systems. This is especially useful with the increasing popularity of wearable sensors [4,5] and the need for smart medical homes for safe aging in place [6,7].

Despite the increased use of context identification techniques in medical systems to build more advanced applications, reviews on context-aware applications in healthcare conducted by Bricon-Souf and Newman in 2007 found that current systems are vastly lab prototypes [8]. Thus, as of 2007, the actual application of context-aware systems in patient populations was minimal, given that most systems were used for research purposes and not for patients/healthcare providers (i.e., lab prototypes) [8]. The other reviews on context awareness in healthcare also primarily reported prototypes, such as the review by Quinde et al. on methods in asthma management [9] and Tobon et al. on context awareness in wireless body area networks [9,10]. A recent systematic review by Gubert et al. was useful for identifying major challenges in the field of context-aware healthcare [11]. However, because the objective of the review was not to report on the current state of context-aware applications in the medical field, systems that are currently being used by patients/healthcare providers were not identified. Additionally, it is unclear what contexts are important for medical context-aware systems and what the different domains of applications are.

The objective of this scoping review is to determine what field-tested context-aware medical systems exist and use them to understand the most common contexts needed in medical systems, as well as what the different categories of context-aware healthcare applications are. We have systematically reviewed the literature and screened for papers that use context-aware systems in conjunction with healthcare providers or patients to provide an overview of the progress made in integrating the context into healthcare applications since the review by Bricon-Souf and Newman.

## 2. Methods

### 2.1. Objectives

The objective of this scoping review is to determine which medical context-aware systems are currently being used by healthcare providers and patients. As this goal is focused on broadly identifying what exists within the literature at present, the review question lends itself well to a scoping review. Additionally, the present work aims to identify which contexts are being used by these systems and to find themes/categories for the context-aware applications that are identified throughout the review. An adapted population, intervention, comparison, and outcomes (PICO) framework for the research question is provided in the protocol attached as Supplementary Materials S1.

### 2.2. Design

Reporting followed established guidelines, and a standard scoping review methodology was used [12,13]. The protocol for this scoping review is attached to the article’s Supplementary Materials S1.

### 2.3. Study Eligibility

Studies that used context-aware technology by either patients or healthcare providers were included in this review. After the level 1 screening of abstracts, a level 2 screening questionnaire was used to rule out studies that included prototypes that were not used by patients or healthcare providers outside of a lab setting. Additionally, the questionnaire was used to exclude systems that did not utilize contextual information to change the end application and thus were not truly context-aware. For instance, Wagner’s initial study on a context-aware blood pressure measurement system was excluded as it did not utilize the contexts collected, such as whether the user had their legs rested for 5 min or was not talking [14]. However, a follow-up study using this system was included since the application changed based on these contexts by telling users not to change their stance or activity (e.g., no talking) based on the collected contexts [15]. Peer-reviewed journal articles were included, and grey literature (conference proceedings or abstracts) and articles not in English were not included in this review.

### 2.4. Search Design and Data Extraction

The following online reference databases were searched: Wiley, ACM, EBSCO, IEEE Xplore, PubMed, ScienceDirect, and SpringerLink. For SpringerLink, an option was not provided to filter for words within the abstract, so a customized filter program was developed in R. It executed the search strategy on the results from the search conducted by their system for keywords present in the full text. These steps reduced the initial article count from 1369 to 111.

The titles and abstracts were independently screened using Rayyan by two reviewers, and all conflicts were resolved prior to the level 2 screening. Articles that passed the initial screening were then included (in Table 1) if 3 questions were answered with YES by each reviewer. Question 1 asked whether the system was used by the patients or healthcare providers. Question 2 asked whether the system was context-aware by changing its application based on context data, and question 3 asked if the system was used outside of a controlled setting (e.g., non-simulated activities outside of a research lab). For studies that passed all three screening questions, standardized spreadsheets (MS Excel) were used to extract general study characteristics, TIDieR checklist items, contexts used within each study, and general information regarding the technology, such as the types of sensors used.

## 3. Results

### 3.1. Study Selection

**Figure 1:**
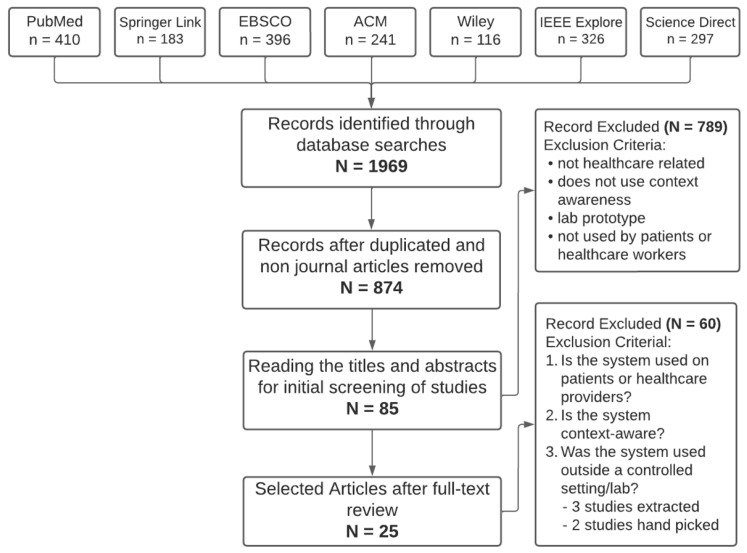
PRISMA diagram of the selection process.

A total of 1969 records underwent title and abstract review, which led to 85 papers reviewed at the full-text level. After a full-text review, 25 papers passed the three level 2 screening questions and were thus eligible. A PRISMA flow diagram illustrating this can be seen in Figure 1. The study characteristics, contexts used, and how the systems were context-aware can be seen in Table 1. The studies were categorized into domains based on a review by Acampora et al. and new domains that were created based on the results of screening [40]. The categories, their descriptions, and papers that fell into each category can be seen in Table 2. In total, 9 out of 25 studies were in the Smart Inpatient/Outpatient Software and Medical Device category, 2 out of 25 in Continuous Health Monitoring, 3 out of 25 were in Assisted Living, 4 out of 25 were in Therapy and Rehabilitation, 6 out of 25 were in Smart Diagnostic and Disease Management Systems, and 1 out of 25 were in Persuasive and Emotional Well Being.

### 3.2. Study Characteristics

Fourteen studies conducted small field tests involving less than 25 patients, eight studies tested their systems in 25–150 patients, and three studies conducted large-scale trials by running the system in either the entire hospital or in more than 1000 patients (Table 1). Thirteen studies implemented the context-aware solution for over one month, and the remaining twelve studies either implemented the technology during a single patient visit or in a timeframe that was less than one month. No pre-existing condition or target population/disease was the sole focus of more than two studies.

### 3.3. Technology and Contexts

Most studies relied on either mobile phones or tablets (10 out of 25), smartwatches (2 out of 25), or integrating a software application into a hospital’s current system (5 out of 25). The remaining studies used other technologies (e.g., virtual reality headsets) or relied on other ambient sensors, such as thermal cameras, infrared motion sensors, or wearable accelerometers. The most common contexts used were user location (8 out of 25 studies), demographic information (6 out of 25 studies), movement status/activity level (7 out of 25 studies), time of day (5 out of 25 studies), phone usage patterns (5 out of 25 studies), lab/vitals (7 out of 25 studies), and patient history data (8 out of 25 studies). Patient history was defined according to the way it was collected in practice (i.e., medical, surgical, medications, allergies, family, and social). The majority of the studies that used patient history information relied on medication history (6 out of 8). Other contexts were more specific to the context-aware systems of interest.

## 4. Discussion

The present work sought to determine the current state of context-aware systems in healthcare relative to Bricon-Souf and Newman’s review in 2007, where it was determined that the majority of systems were still lab prototypes [10]. In the decade following this review, various research teams have managed to develop functional context-aware systems that have been tested in healthcare environments via their use in patients and by healthcare providers. Most of these applications are still in their early stages and have been used by less than 150 people in a brief field test. The notable exceptions are the system implemented by Borbolla et al., where half of the patients in the hospital were able to view information explaining medical tests/disease specific to their current context, and the drug–drug interaction system built by Cornu et al., which was tested in a 721-bed hospital [26,31]. We used the results of the search in conjunction with the ambient intelligent medical application categories proposed by Acampora et al. to develop the six domains for context-aware healthcare systems shown in Table 2 [40]. Although Acampora et al.’s categories were formed for ambient intelligent systems, context awareness is a pivotal requirement of ambient intelligent systems [40]; thus, there is much overlap between these systems and their application categories. Evidently, the smart inpatient/outpatient software and medical device category, which uses context-aware systems within hospitals and clinics to improve patient care, has had the most success in reaching healthcare providers and patients (9 out of 25 studies). The second most prominent category in terms of the number of applications being used by patients/healthcare providers is the smart diagnostic and disease management systems (6 out of 25). Following this category are assisted living applications (4 out of 25) and therapy and rehabilitation applications (2 out of 25). We chose to create subdomains when appropriate, such as further dividing smart diagnostic and disease management systems into diagnostic systems and disease management systems. However, in the future, if major differences in required contexts are discovered, it may be more logical to divide many of these subcategories into their own domains.

### 4.1. Smart Inpatient/Outpatient Software and Medical Device

The smart inpatient/outpatient software and medical device class of context-aware applications aim to improve the quality of life (work efficiency, measurement accuracy, etc.) of hospital stakeholders. These stakeholders can be patients, doctors, nurses, staff, or anyone else working at the hospital. Many of these applications solely rely on software to help these stakeholders; however, some interesting applications have emerged that use smart equipment that functions primarily in a clinical setting to provide medical information on patients to healthcare providers or to simplify procedures performed by providers by integrating contexts.

Smart hospital applications of context-aware systems are likely the earliest use cases of context awareness in medicine and seem to be the most prevalent source of context-aware applications to date. The earliest context-aware application identified in this review, dating back to 2011, was a navigation system created by Kim et al., which was used to direct patients within a hospital [16]. In this study, each patient was given a tablet with an app that showed them how to navigate to their final destination, what to do at that location, and communicated with the hospital’s information system to know when they completed a task and should be directed to their next required task [16]. Regarding smart equipment, one field-tested application developed by Lindahl et al. involved a context-aware smart chair equipped with a blood pressure cuff/monitor to facilitate BP measurements in pregnant women at their 12-month ultrasound appointment. The purpose of this system was to diagnose hypertensive diseases and preeclampsia [15]. The primary contexts used by the system were the users’ position/state during the BP measurement, including whether their legs were uncrossed, whether their back was against the chair, and whether they were resting or talking. An interesting result shown in the study was that when resting and not talking were enforced by the system and legs/back position were not enforced, the compliance for the enforced activities was more than 20% higher than that for the non-enforced activities. This shows how a context-aware system can improve medical measurements by providing users with feedback on what is needed for the system to obtain proper medical measurements. Researchers are also working towards integrating context into operating rooms, as shown by the system by Franke et al. [41]. This system was not field tested and thus was not included in our final study list. Instead, the system was tested on 24 recordings of real surgical operations and showed how the current context of the surgery (e.g., the next step in the procedure) can be used to predict what the surgeon would like to do next and adapt the equipment settings and hospital software accordingly. Some examples include automatically determining the billing code based on the procedure, changing the lighting of the endoscope based on the current image, automatically switching to navigation displays whenever the pointer is being used, and reducing the force of the surgical equipment near sensitive structures [41].

One final emerging area of application in smart hospitals is the analysis of a patient’s specific context via their electronic medical records to detect possible errors. One application of this that has been field tested is looking at patients’ lab values and drug prescriptions to decide whether the drug dosage is incorrect or the drug itself should not have been prescribed given the patient’s current kidney and liver function. Niazkhani et al. used clinical guidelines regarding drugs prescribed by nephrologists at a kidney transplant clinic to determine the proper dosages and prescribing rules given a patient’s specific context, such as their kidney function, liver function, pregnancy status, and other demographic data [33]. Their system was then field tested in 100 patients and used these rules to alert physicians when problematic drug lab interactions (DLIs) existed, of which 260 DLIs were found [33]. The largest field-tested study identified was a similar system tested in a 721-bed hospital over 14 months developed by Cornu et al. that used contexts such as patients’ current medications, age, sex, last potassium levels, and renal function to develop clinical decision rules that warn physicians about dangerous drug–drug interactions [31]. Similar systems are being worked on for general drug monitoring in the elderly, illustrating that this context-aware healthcare domain is a highly active research area [42].

### 4.2. Assisted-Living

Assisted-living applications of context-aware systems primarily focus on supporting patients and the elderly during their daily activities to facilitate independent living within their primary residences and improved quality of life. For instance, an application that may be highly beneficial in those living with cognitive impairments due to neurological diseases (e.g., dementia) is context-aware medication reminder systems. These systems, which could be used to remind patients of activities other than taking their medications, attempt to understand a user’s current context to optimize the chance that they will see a reminder and act on it. An excellent study that was field tested in 10 users over 28 weeks by Hayes et al. demonstrated the efficacy of this approach by comparing the results when users underwent 10 weeks without prompts, 10 weeks with prompts, and 10 weeks with context-aware prompts [28]. The context-aware prompts used motion sensors to detect where a user was in their home and then sent a visual/audio prompt to a beacon in that room, as well as a message to their smartwatch, when the user should take their meds. However, if they were in bed (bed sensor) or on the phone, already took the med from the pill box, or were not at home (contact sensor), the prompts were not sent. Additionally, prompts were only sent within 90 min of when they should have taken the meds. Context-based prompting resulted in significantly better adherence (92.3%) compared to time-based (73.5%) or no-prompting (68.1%) conditions (*p* < 0.0002, χ^2^ = 17.0) [28].

Another class of assisted-living applications is those that focus on managing patients’ pre-existing diseases. The best field-tested example of a system that focuses on this is the smartphone-based self-management system developed by Ong et al. for chronic kidney disease (CKD) [34]. In this system, patients were given a smartphone with an application that was linked to their BP recording device. They were asked to answer questions about their symptoms and medication changes regularly, and this information was shared with the care team to facilitate continuous monitoring. If dangerous medication changes existed, or symptoms worsened significantly and warranted intervention according to clinical guideline-based rules, then the care team received an urgent update. Feedback on BP control was provided to patients via the application, and the application would make recommendations depending on the patient’s context/circumstances. For instance, if the patient had elevated potassium levels, dietary modifications would be recommended [34]. The final results of the study were quite compelling as the mean systolic and diastolic blood pressure of the 47 patients decreased by 3.4 mmHg and 2.2 mmHg, respectively.

### 4.3. Smart Diagnostic and Disease Systems

Smart diagnostic systems focus on determining a patient’s condition/disease in the absence of physicians, and context-aware disease management systems help patients manage their user conditions according to the contexts surrounding their current disease state. This includes applications based on sensor systems that aim to help reduce the symptoms and issues present in patients living with chronic diseases. A good example from Yin et al.’s review of context-aware systems for chronic disease patients is the wearable system developed by Bachlin et al. to assist those with Parkinson’s disease (PD) during walking [21,43]. Given that evidence suggests that rhythmic auditory stimulation can help Parkinson’s patients move when they are stuck due to freezing of gait (FOG), Bachlin et al. developed a system that detects FOG in real time so that they can then provide audio cueing to assist patients. In this case, the context is the patient’s gait status according to the accelerometer data recorded from the patient’s knee and ankle. Although the system was not proven to improve FOG in their small test of 10 PD patients, physiotherapists believed that the system was helpful [21].

Coronata et al.’s study is an example of a system that is used for diagnostic purposes not to diagnose a disease but to detect motion disorders for those with an autism spectrum disorder. This was performed by training an artificial neural network on accelerometer data and using contexts such as the time of day and duration of the gestures. The system was used on five patients within a hospital, and the online classifier achieved an accuracy of 92%, showing that there is promise for the approach [18]. Another application of context awareness in disease management is the system by Dai et al., who improved prostate segmentation during image-guided radiation therapy using patient-specific contexts obtained from their prior images. The system was tested on 24 patients, and they found that using prior personalized image data led to improved prostate segmentation accuracy, as defined by the dice ratio and average surface distance [30]. As disease management is quite specific to the condition of interest and the patient’s ability to follow what are often complex guidelines, context-aware disease management systems have the potential to improve patient health by helping them complete the necessary daily tasks required to manage their disease. Furthermore, it may reduce emergencies like asthma exacerbations, as systems like Kaffash-Charandabi et al.’s use of environmental contexts to predict possible hazardous conditions for patients to guide them to avoid scenarios that can lead them to the emergency room (asthma exacerbation in this case) [19].

### 4.4. Therapy and Rehabilitation

The therapy and rehabilitation category of context-aware healthcare systems focuses on systems that provide psychology-based therapy to improve mental illness or physical rehabilitation for people with conditions suspected to benefit from physiotherapy. One interesting therapy-related system developed by Stratou et al. was field tested in 100 patients and demonstrated promising results for determining the effect on patients during psychological interviews [37]. Their camera-based system investigated the users’ eye contact, smile level, and other behavioural indicators to determine their distress levels. An example of the context used by the system is what the users’ affect should be based on the topic of conversation. For instance, if asked to describe a recent positive event in their life, and then asked to describe a negative event, the system would expect a smile during the first story and a less positive facial expression during the second question. The results were promising as the team was able to show that the models that considered context variables had a much better correlation with post-traumatic stress disorder and depression scores in 100 patients taken from the general and US veteran populations [37].

The present work only identified one rehabilitation-related context-aware system that was field-tested in patients and utilized context. In the virtual reality application identified in the search, which was tested in users with epicondylitis, the difficulty of arm exercises that utilize the VR system controller and the chosen activity was recommended based on the users’ current progress. Users were instructed to perform 20 exercises with their arms, 10 of which were conducted with a virtual weight and 10 with the user holding a real weight while using the VR app. Although the clinical benefits were not assessed, it was demonstrated that the time taken to complete the tasks and the amount of deviation from the optimal trajectory decreased as users went from their first session to their last. Integrating user-specific performance/context into rehabilitation applications will likely be an important development in personalizing programs for patients to improve their mobility, especially when physiotherapists are not present to correct imperfections in the patient’s form during the exercise [23].

### 4.5. Continuous Heath Monitoring and Persuasive and Emotional Well-Being

We defined continuous health monitoring as systems that constantly track the physiological data of patients (e.g., vitals, blood glucose, etc.). Additionally, we removed activity monitoring from this category relative to Acampora et al., given that most behavioural monitoring applications usually monitor behaviour to use it to build applications in other categories, such as assisting the elderly in their home (assisted living) [27,28,34], delivering psychology-based therapies (therapy and rehabilitation) [32], and providing emotional support (emotional well-being) [29]. Our screening captured minimal continuous health and activity monitoring applications that are currently being used by patients and healthcare providers. In reality, there are likely many emergency and fall detection systems that use basic contexts like immobility, fall sounds, and rapid altitude or acceleration changes to detect falls or emergencies [44,45]. However, these systems do not focus and thus do not mention context awareness. Thus, by design, they were not captured by our search criteria since the present work focuses on systems that intentionally integrate context awareness. One interesting application was captured that uses a non-contact method to determine the respiratory rates of people as they sleep. Near-infrared cameras assessed the users’ body context, and if it was below a threshold representing a still user, the system focused on the users’ subtle remaining motion (e.g., chest during breathing) to determine their respiratory rate. The results were promising, as the correlation with another commercially available system (CO2SMO PLUS) was 0.9 [20]. Another continuous health-monitoring study focused on using contextual data to better understand the ECG patterns of patients. For instance, in the case studies, they were able to rule out a false positive tachycardic event by recognizing that a heart rate of over 100 was normal given that the user was running [38].

Limited studies were found on the persuasive and emotional well-being-based context-aware systems that are currently being used by patients or healthcare providers. This too is likely due to a lack of emphasis on understanding context awareness techniques in healthcare applications that focus on persuading people to make better physical (e.g., diet) and mental health (e.g., meditation apps) choices. We have defined a primary difference between persuasive and emotional well-being applications and therapy applications in the other domain to relate to how the former is readily available to the public and does not only focus on improving the mental health of those with known psychiatric-related issues. Wahle et al. developed a mobile app that any individual can download and use. The app recommends personalized interventions to help reduce depression levels. Contexts such as the user’s calendar events, walking time, time at home, and number of calls were used to predict their depression levels. The results were promising as a significant reduction in PHQ-9 depression scores from the initial questionnaire was found for those with a clinically relevant baseline, and they were able to predict scores above a threshold better than a random binary classifier [29].

### 4.6. Important Contexts and Technology within the Domains

As each of the six categories of context-aware healthcare systems aims to solve a different medical challenge, the contexts and technologies utilized within each category seem to differ (Table 1). However, there seem to be a few pivotal contexts that are present across domains that most context-aware medical systems leverage. User location, time of day, and whether the user is in an active or resting state appear to be three important contexts in many applications. This is an intuitive result as the user’s location and time of day often dictate what the application should do. For instance, a nurse may not be sent a low-priority pager request in the operating room if the time of day aligns with a patient’s operation or a patient consult [36]. Additionally, many applications rely on user activity levels to understand whether notifying them to do something is likely to be accepted or not [28,32]. These three contexts are likely to be significant in any context-aware application involving users. Groups such as Alti et al. have begun to create ontology models for context-aware healthcare applications that formally define some of these important contexts. For instance, their seven main sub-contexts are the “UserContext”, “PlaceContext, and “ActivityContext”, which similarly define user details, their location, and activity, respectively. The other four sub-contexts are the “ResourceContext” describing system resources (e.g., processing power), the “HardContext” defining the devices that the smart services execute on, the “DocumentContext” specifying the properties of relevant media files, and the “BiomedicalContext” that gathers context data pertaining to the user’s health [46].

Regarding health-related contexts that seem to span multiple health categories, a user’s past medical history (e.g., medications, illnesses, and past surgeries), demographics (age, sex, and weight), and lab values seem to be prevalent across categories. Regarding contexts within each subdomain for smart diagnostic and disease management systems, the important contexts aside from past medical history seem to be unique to the disease of interest, such as pollutant levels in Kaffash-Charandabi et al.’s asthma application and the movement types in Coronata et al.’s motion disorder detection system [18,19]. For assisted living applications, the user’s current activity, in conjunction with location and time of day information, seems to be a key context for understanding whether it is a good time to interact with them (e.g., the cue to take medicine). Disease-specific contexts are also used to understand what the application must do (e.g., cue BP measurements) and when to intervene (e.g., BP over 140 regularly). Too few therapy and rehabilitation applications were found to confidently comment on the important contexts. However, the user’s current performance during exercises and whether the user has a low cognitive load at that time and is thus available for therapy-related interventions seem to be a promising domain-specific context. Too few continuous health monitoring and persuasive/emotional well-being applications were found to confidently comment on the domain-specific contexts as well. However, continuous health monitoring will presumably leverage contexts extrapolated from vitals/physiological parameters, (e.g., tachycardia) and persuasive/emotional well-being will likely rely on contexts that help understand the users’ activities and availability for prompting them to engage in applications that push towards better lifestyle choices.

In addition to the contexts varying between categories, the technology implemented also varied, with some categories relying on software versus hardware. For instance, smart inpatient/outpatient applications mostly rely on software to improve workflow efficiency in current clinical settings. In this case, smart devices mostly relay data to servers to better understand patient measurements, although the potential for hardware to help ensure proper measurement conditions has been shown [15]. Smart diagnostic systems will likely rely on wearable sensors, whereas disease management systems may rely on software more to understand how a user is managing their disease based on their symptoms and self-reported measurements via an application. We have defined continuous health monitoring applications as those that constantly obtain medical data and ensure that they are within normal bounds, so these will presumably rely on wearable sensors and software applications that cue users and HCPs if anomalies are detected. Assisted-living applications seem to rely on Internet of Things (IoT) devices and smartphone sensors to understand a user’s context and determine when to cue them to do things like taking their meds or completing something else that is required to manage their disease (e.g., BP measurement high blood pressure). Rehabilitation applications will likely rely on wearable sensors paired with software to encourage and guide users through exercises, whereas therapy-related applications will likely rely more on software to guide and encourage users via evidence-based treatments. Lastly, we predict that persuasive/emotional well-being applications will mostly leverage smartphone or smartwatch sensors and software to encourage people to make good decisions when they seem to be under low cognitive load and thus available for prompting.

## 5. Limitations and Future Research

Looking at the date of the applications found in this review, it appears that all applications found to be in use within patient populations or used by healthcare providers were published after Bricon-Souf and Newman’s review of context-aware healthcare systems in 2007 [10]. Thus, whereas the field of context-aware medical systems was mostly in its conceptual phase until 2007, it appears that it is now in its developmental phase. Most of the current applications focus on smart hospitals, assisted living, and helping users manage their medications/diseases. Few continuous health monitoring and persuasive/emotional well-being applications were found in this review, which likely reflects the lack of current applications emphasizing context awareness rather than the actual state of context-aware systems in this application class.

After assessing each paper for its context determination method, it became apparent that most context-aware systems currently used by healthcare providers do not have a general method of quantitatively determining the likelihood of each context. This highlights the interoperability challenges with context-aware systems, as the way a context is identified and used by one system will likely differ from that of the next system. In fact, few (if any) papers have described a general taxonomy for context-aware systems, which may be needed given that there are many components to these systems (e.g., data acquisition, pre-processing, context-collection, system adaptation to contexts, etcetera). One such example of a general taxonomy can be seen in the work of Perera et al., where they reviewed 50 context-aware IoT systems to develop a taxonomy where contexts undergo acquisition, modeling, reasoning, and distribution among the context-aware systems [47].

Many papers have relied on machine learning methods, which would require the inputted data to be similar when using interoperable data across systems [23,30]. That being said, manual labelling of training data to generate significantly large high-quality datasets to create models that predict new contexts may not be practical at scale or when trying to infer many everyday contexts. Obtaining quality data may be further complicated by privacy concerns surrounding users’ health data and security concerns when transferring their data across networks or storing them in cloud databases [48]. Lastly, systems targeted for medical grade applications are often costly, and in addition to being costly, context-aware medical systems may be difficult to scale given the aforementioned data requirements. However, it appears that a sub-class of consumer-oriented context-aware software applications (e.g., personalized mobile apps for depression monitoring [29]) may not suffer from these cost and scalability problems by leveraging hardware from smartphones and user inputs for training data [19,22,24,25,29,34].

The identified limitations and their presence in many of the extracted studies lead to gaps in context-aware systems, which should be addressed through future research. The field may benefit from future research directed towards integrating context-aware privacy/security frameworks that are in development by other groups into currently active systems [49]. Additionally, a major gap identified was the lack of consistent context-determination methods across the extracted articles and often limited details regarding how the contexts were extracted. Bridging this gap and using one of the general frameworks that researchers have been working towards for context-aware systems may help field progress [47]. This could help systems interoperate in the future and share context-determination algorithms (when systems have overlapping sensors). Given that there may be thousands of useful contexts surrounding user activities in their environments, this is likely a more practical long-term solution than each system attempting to collect the necessary data to identify every possible context.

## 6. Conclusions

Context-aware healthcare applications have finally begun to reach healthcare providers and patients. Contexts, such as user location, time of day, patient demographic data, and medical history, have been pivotal to the success of these early applications. Additionally, different applications have different contextual requirements. The present work set out to leverage the information in these early applications to better understand the contexts needed to build different healthcare applications. Hopefully, with this better understanding of the key contexts used within various subdomains of context-aware healthcare systems, researchers can leverage these findings to ensure that their systems contain contexts that were useful in the early applications identified in the present work.

## Figures and Tables

**Table 1:** Study Characteristics and Context Selection/Use.

Article	Year	General Description	Sample Size	Mean Age	Test Length	Measured Signal	Assessment Method	Contexts Used	Context Awareness (How Did the App Change)
Patient Satisfaction in a Context-Aware Hospital Guidance System [16]	2012	7.1-inch Galaxy Tablet with the hospital guidance system app [16]	13	44	Hospital visit	Patients’ location within the hospital	Patient satisfaction survey	Patient location within hospital, appointments, and procedures the patient needs to complete	The application changed based on the patients’ specific next task in the hospital; guidance changed based on their current location
A Hospital Bed Allocation Hybrid Model Based on Situation Awareness [17]	2018	Web app for hospital bed allocation [17]	50	N/A	5 days	Rate of successful placement	Verify whether the bed selected was correct	Room type based on patients’ health plan, physician specialty, sex, type of treatment, risk (e.g., infectious), degree of dependency, age, time of hospitalization	Bed displayed to bed manager varied based on patient-specific context
A situation-aware system for the detection of motion disorders of patients with autism spectrum disorders [18]	2014	Wristwatch with an accelerometer worn by patients [18]	5	N/A	N/A	Detection of movement disorders	Compared with ground truth	Hand gesture type, time of day, gesture duration	The clinical reports varied based on contexts such as the duration of each gesture, time of day of the gesture, and type of movement disorder
A ubiquitous asthma monitoring framework based on ambient air pollutants and individuals’ contexts [19]	2019	Smartphone with a context-aware asthma management app [19]	3	36.33 (32F, 35M, 42M)	N/A	Predicted peak expiratory flow (PEF)	Compared predicted PEF to actual PEF from devices that patients used	Environment/pollutant variables, user location, user’s age, gender, height	App warns patients of potential asthma attacks via predicted PEF which changed based on various contexts (e.g., environment/pollutant variables, user location, user’s age, gender, height, and PEF)
A Visual Context-Awareness-Based Sleeping-Respiration Measurement System [20]	2010	Near-infrared camera that monitored users as they slept to determine respiratory rate [20]	18	N/A	N/A	Respiratory rate	Compared RR results to those of the CO2SMO PLUS respiratory monitoring machine	Body motion of users	The system determining respiratory rate changes its action based on the body motion context (proceeds to RR calculation if still)
A wearable system to assist walking of Parkinson’s disease patients [21]	2010	Accelerometers used to detect freezing of gait (FOG) context in Parkinson’s in real time [21]	10	66.4	237 FOG events	Freezing of gait	Physiotherapists observed video to determine FOG events and detection by the system was compared to ground truth	Gait state (i.e moving vs. frozen)	Auditory stimuli delivered if FOG context detected
CARE: Context awareness for elderly care [22]	2020	Android mobile app that shows activities of older adults determined by sensors in retirement homes [22]	15 patients, 17 nurses	N/A	2 months	Long-term trends in resident activity level, time in bed, proximity to nurses, proximity to other residents	Questionnaire was used to determine if nurses found the application useful	User location, nurse location, time in bed, level of activity	Different data displayed to nurses via android app depending on user contexts (e.g., how often resident is near nurses or other residents, their level of activity, etc.)
Connected Elbow Exoskeleton System for Rehabilitation Training Based on Virtual Reality and Context-Aware [23]	2020	VR-based physiotherapy application paired with an elbow exoskeleton device [23]	5	N/A	1 month	Performance of patient over time in VR rehab exercises where patient moves ring along cable, but the ring should not touch the cable	Position, angle, and time deviation during the exercise relative to a perfect performance (e.g., wire kept in the center of the ring for position performance)	Performance on exercise	Patients’ performance on current exercise is used to inform how difficult the app makes the next exercise
Effects of context-aware patient guidance on blood pressure self measurement adherence levels [15]	2019	Smart chair and tablet paired with a blood pressure (BP) monitor to ensure proper contexts for BP measurement [15]	100	29.9	One time	Adherence to the requirements for proper blood pressure measurements when recommended by the system versus not recommended	Percent adherence to the different requirements	User rest time, legs crossed, back-supported, ambient noise/talking, participant compliance	App recommended rest time and not talking if motion/talking contexts were detected; other contexts not recommended
Evaluation of an optimized context-aware clinical decision support system for drug-drug interaction screening [24]	2021	Drug–drug interaction intervention application in hospitals [24]	2630 alarms	N/A	8 months	Drug–drug interactions and the acceptance rate of alerts	Tracked numbers of alerts that result in a change to a prescription	Patients’ current medications, age, sex, last potassium levels, and renal function	Application utilized patient-specific data to determine if it flags the current prescription as dangerous
Harnessing Context Sensing to Develop a Mobile Intervention for Depression [25]	2011	Mobile application to improve and predict major depressive order [25]	8	37.4	8 weeks	Mood, location, activity, who the users were with or near	Compared predictions to those entered manually by participants	Location, time of day, who users were with, conversing or not, mood, contexts from phone apps such as recent calls, active applications	Moods predicted and thus tracked/displayed to users changed based on machine learning models that predicted mood from context data derived from phone sensors
Integrating Personalized Health Information from MedlinePlus in a Patient Portal [26]	2014	Patient portal in hospital that provides medical info specific to the patient’s context [26]	80,000	N/A	1 year	Use of the lab test info and MedlinePlus explanatory info buttons	Number of clicks and percent of sessions the buttons were used in	Patients’ lab test results and condition/disease	The information offered to patients viz the patient portal varied based on what disease they had, or lab tests they received
MHS: A Multimedia System for Improving Medication Adherence in Elderly Care [27]	2011	Pill station equipped with a webcam that registers new medications and devices that prompt users to take meds [27]	5	Over 60	3 weeks	Adherence to taking medication	Unclear how ground truth medication taking is established, but likely assumed they take the medication when a pill box is opened	Users’ current activities such as watching TV, talking on the phone, eating, sitting, moving; exact list of used contexts not clear	The application prompts users to take medication based on their current contexts; i.e., if a pill is required with a meal, then they are prompted based on the eating context
A Study of Medication-Taking and Unobtrusive, Intelligent Reminding [28]	2009	Medication reminders of auditory/visual cues at user’s location in their home [28]	10	82.7	10.7 weeks	Adherence rate for taking medication	Pill recorded as taken when the smart pill box container is opened	Location in home, when they are leaving home, a phone call in progress, in bed, using laptop, preferred time for medication	Audio and visual prompts around the home were triggered based on contexts, such as the user’s location, if they are not on the phone, the normal time they take the pill, if they have not taken the pill, if they are about to leave home
Mobile Sensing and Support for People With Depression: A Pilot Trial in the Wild [29]	2016	Mobile app for depression intervention and depression scores [29]	126	20–57	Over 9 months	PHQ-9 depression score	Compared PHQ-9 scores obtained from patients through questionnaire over time	Time of day, location, smartphone usage, activity level, walking time, time at home, geographic movement, number of unique Wi-Fi fingerprints, number of calls, calendar events	Interventions recommended varied based on users’ context (e.g., low activity level, walk recommended) and users’ feedback on interventions
Online updating of context-aware landmark detectors for prostate localization in daily treatment CT images [30]	2015	Prior treatment images used to improve landmark detection and prostate segmentation [30]	24	N/A	Length of treatment	Accuracy in segmenting prostate volume for algorithm relative to segmentation by physicians	Dice ratio and average surface distance	Patients’ inter-landmark distance, intra-landmark distance, and prostate segmentation from their prior images	The algorithm that identified landmarks and performs prostate segmentation varied based on patients’ prior treatment images and any adjustments from the physicians on the landmark/segmentation
Pilot evaluation of an optimized context-specific drug–drug interaction alerting system: A controlled pre-post study [31]	2015	Rule-based software applications to prevent dangerous prescriptions [31]	1116	N/A	14 months	Acceptance rate of alerts when a new system that included context data was implemented	Tracked number of alerts that results in a change to a prescription	Patients’ current medications, age, sex, last potassium levels, and renal function	Application utilized patient-specific data to determine if it flags the current prescription as dangerous
Prompto: Investigating Receptivity to Prompts Based on Cognitive Load from Memory Training Conversational Agent [32]	2020	E4 wristband determines users’ availability for memory training [32]	7	67.4	1 week	Responses to prompts and appropriateness of prompt timing according to user feedback	Percent of prompts that were accepted for memory training to commence	Cognitive load of users determined via heart rate variability and electrodermal activity	Application prompted users for memory training when cognitive load measured by E4 wristband was low
Translation of evidence into kidney transplant clinical practice: managing drug-lab interactions by a context-aware clinical decision support system [33]	2020	Smartphone application that prompts CKD patients to take BP measurements and symptoms [33]	100	47.44	N/A	Clinician satisfaction with the system	The “Questionnaire for user interface satisfaction”	Renal function via creatine clearance, lean body weight, pregnancy status	The system generated drug–lab interaction alerts based on the patient’s specific lab values (creatine clearance), lean body mass, and pregnancy status
Integrating a Smartphone–Based Self–Management System into Usual Care of Advanced CKD [34]	2016	Mobile app focused on predicting stability of bipolar disorder patients [34]	47	59.4	6 months	User satisfaction with the app, change in blood pressure, change in CKD-relevant lab values	Exit interviews to assess satisfaction, BP measured by home monitoring device, and results compared between baseline and end of study	Patients’ adherence to BP measurements, their symptoms, and their medications	Frequency of messaging patients for BP changes based on their compliance, alerts sent to healthcare providers if symptoms warrant it, and medication discrepancies checked by the system throughout
Automatic detection of social rhythms in bipolar disorder [35]	2016	Social and activity contexts inferred from smartphone sensor data are used to predict social rhythm metrics [35]	7	4 users 25–34; 3 were 34–64	4 weeks	Social rhythm metric	Compared SRM from models to that determined by manual inputs from patients	Phone usage patterns, location, distance traveled, number of conversations per day, duration of conversations, time speaking to others, speaking rate, speech pitch, time active vs. sedentary, SMS/call activity	Inferred behavioural rhythmicity and SRM changes based on contexts measured
Alarm Fatigue vs. User Expectations Regarding Context-Aware Alarm Handling in Hospital Environments Using CallMeSmart [36]	2017	Handheld communication system considers healthcare providers’ current context/activity to understand whether to page the user [36]	N/A	N/A	2014–2017	Satisfaction of healthcare workers with the new system relative to the old system	Interviews with users	Users’ calendar events (e.g., no calling while in patient consult), location (e.g., operating room)	Messages are relayed to healthcare workers based on urgency and their current availability as determined by their context (e.g., unavailable during patient consult)
MultiSense—Context-Aware Nonverbal Behavior Analysis Framework: A Psychological Distress Use Case [37]	2017	Topic of the discussion and what a normal, positive, or negative response looks like is used as context to determine distress levels [37]	100	N/A	One interview session	Distress levels of the person being interviewed	Root mean square error in systems predicted distress levels relative to ground truth levels	Users’ eye contact, smile level, and other behaviour indicators along with what the users affect should be based on the topic of conversation (e.g., smile while describing a trauma atypical)	Applications use patients’ non-verbal behavioural contexts to predict their distress levels and generate a patient-specific report
mCardia: A Context-Aware ECG Collection System for Ambulatory Arrhythmia Screening [38]	2022	Mobile electrocardiogram system uses contextual data in free-living conditions to provide a better understanding of what happens before, during, and after an arrhythmia [38]	24	58.79	2 weeks	Electrical activity of the heart by the ECG	Participants were explained what was considered an event and taught how to report these events through the app	Gender, age, height, weight, sensor location, body position, step count, metabolic level, activity level	Would not consider high heart rates dangerous if the context warranted a higher heart rate (e.g., running)
Design and usability evaluation of COOK, an assistive technology for meal preparation for persons with severe TBI [39]	2021	A stove and kitchen are equipped with various sensors to assist those with past traumatic brain injuries in cooking [39]	3	Between 39–57	6 months	Heat surrounding the stove and whether human activity is present	User satisfaction according to questionnaires and evidence of increased stove use when cooking meals	Stove on or off, apartment vacant, apartment occupied, cooking, absent from the kitchen	The system warns the user if a dangerous situation/context is detected, such as the stove is left on after cooking; it may also turn the stove off itself

**Table 2:** Application categories and their contexts.

Application Category	Description	Subcategories	Reference Number	Contexts Found	Important Contexts	Technology Descriptions
Smart Inpatient and Outpatient Software and Medical Devices	Software systems to improve communication between healthcare staff and patients within the hospital	(A) Hospital/Inpatient systems (B) Outpatient systems	(A) [16,17,24,26,31,33,36] (B) [15,22]	healthcare provider location *, Patient location *, appointment time *, procedure type *, age *, sex *, physician specialty, medication list, lab test results, renal function *, weight, user taking or not *, activity level, time in bed *	location, calendar, medical history, medication list, demographic info,	Software applications using expert systems or machine learning and known contexts to make decisions; smart equipment using context to improve medical devices
Smart Diagnostic and Disease Management Systems	Diagnose patients’ using algorithms and identify ideal treatment plans	(A) Diagnostic systems (B) Disease management	(A) [18] (B) [19,21,25,30,35]	Movement type/status *, time of day *, age *, sex *, height, medical history, location *, who user is with *, phone use *, conversing or not *, medical images, disease-specific contexts (e.g., pollutant levels asthma)	Lab results, medical history, demographic data, time of day	Wearable sensors, machine learning, and user input used to make diagnoses and manage the disease
Continuous Health Monitoring	Wearable and ambient sensors for continuous healthcare monitoring	(A) Longitudinal physiological data monitoring (B) Emergency detection	(A) [20,38] (B)	Motion of the user’s body	Location, time of day, vitals, medical history, lab results	wearable sensors/medical devices to understand patients’ health and contexts like past medical history used to determine what normal is physiologically
Assisted Living	Developing smart environments to assist patients in their daily living activities	(A) Disease tracking (B) Physical Support (C) Social Support	(A) [27,28,34] (B) (C) [39]	User current activity (e.g., eating, tv), location *, conversing or not *. time in bed *, medication list *, symptoms, adherence to taking measurements (BP)	IADLs, location, time of day, symptoms, medication list	Mobile, web apps, and IoT devices used around the house to understand daily activities to help users perform tasks (e.g., taking medication) and manage disease
Therapy and Rehabilitation	Providing psychology-based therapy to improve or heal a disorder	(A) Smart rehabilitation (B) Psychology-based therapy	(A) [23,37] (B) [32]	Current performance in rehab tasks, cognitive load	Body position, mood	Wearable devices or software used to guide therapy or provide outputs that can be used in therapy
Persuasive and Emotional Well-Being	Systems aimed at improving physical and mental well-being	(A) Emotional analysis/state systems	(A) (B) [29]	Location, time of day, who users are with (alone, friends, family), conversing or not, mood, contexts from phone apps such as recent calls, active applications	Mood, activities, users’ specific goals	Mobile and web app software leverages smartphone sensors and IoT devices to understand the users’ state and lead them to better lifestyle choices

* Context found in more than 1 study.

## Data Availability

Data sharing not applicable as no new data was created. Supplementary Materials contains the protocol for the review and search terms for each database.

## Supplementary Materials

The following supporting information can be downloaded at: https://www.mdpi.com/article/10.3390/ijerph20146399/s1, Supplementary Materials S1: The protocol for the review and search terms for each database, [12,50,51].
