# Supplementary material for: Context-Aware Medical Systems within Healthcare Environments: A Systematic Scoping Review to Identify Subdomains and Significant Medical Contexts
Source: Int J Environ Res Public Health. 2023 Jul 19;20(14):6399. doi: 10.3390/ijerph20146399 (PMC10379857; doi:10.3390/ijerph20146399)
Supplement: Supplementary file 1 [file ijerph-20-06399-s001.zip › ijerph-2353143-supplementary.pdf]

1. **Review title:**

   Context Aware Computing Systems in Healthcare: A scoping review protocol

2. **Anticipated or actual start date:**

   26 April 2021

3. **Anticipated completion date:**

   July 5 2023

4. **Stage of review at time of this submission:**

   Completed

5. **Named contact:**

   Michael Zon

6. **Named contact email:**

   zonm@mcmaster.ca

7. **Named contact address:**

   1280 Main St W, Hamilton, ON L8S 4L8

8. **Named contact phone number:**

   905-525-9140 ext. 24227

9. **Organizational affiliation of the review:**

   McMaster University

10. **Review team members and their organisational affiliations:**

Title	First Name	Last Name	Affiliation
	Michael	Zon	Department of Medicine, Faculty of Health Sciences McMaster University, Hamilton, Ontario, Canada<br>Department of Biomedical Engineering, Faculty of Engineering, McMaster University, Hamilton, Ontario, Canada
	Guha	Ganesh	

Department of Biomedical Engineering, Faculty of Engineering, McMaster University, Hamilton, Ontario, Canada

Dr. Qiyin Fang

Department of Engineering Physics, Faculty of Engineering, McMaster University, Hamilton, Ontario, Canada

Department of Electrical & Computer Engineering, Faculty of Engineering, McMaster University, Hamilton, Ontario, Canada

School of Interdisciplinary Science, Faculty of Science, McMaster University, Hamilton, Ontario, Canada

11. Funding sources/sponsors:

McMaster Institute for Research on Aging (MIRA) and Natural Sciences and Engineering Research Council of Canada

12. Conflicts of interest:

There are no known conflicts of interest.

13. Review question(s):

The objective of this scoping review is to determine what medical context aware systems are currently being used by healthcare providers and patients. As this goal is focused on broadly identifying what exists within the literature at present, the review question lends itself well to a scoping review. Although a review of context aware systems in healthcare has been conducted in the past, systems that are currently being used by patients/healthcare providers were not identified. This is likely largely due to a lack of applications past the prototype stage at the time of the last review.

A secondary objective is to identify which contexts are being used by these systems and to find themes/categories for the context aware applications that are identified throughout the review. An adapted PICO framework for the research question is provided below.

14. Literature search:

Standard scoping review methodology will be used and reporting of results will follow established guidelines.[1, 2] A systematic literature search will be piloted on Medline using the broad search concepts of “context-aware*”, “health”, and “patient”, with relevant key terms nested under each concept. The process of search design will be iterative. Over a series of piloting cycles, a workable final search will be obtained that will be adapted to other search databases. Searches will be assessed based on whether they yield a hand-selected list of key studies that are deemed to fit the research question. The databases that will be searched are SpringerLink, EBSCO, PubMed, IEEE Xplore, Wiley, ScienceDirect, and ACM. The search interval was from the earliest data available on the

database to May 2021 and only published peer reviewed journal articles were considered. This was done to ensure only quality systems and study designs/methods that have been reviewed by experts are included, thus giving a more accurate representation of the state of context aware systems in healthcare. Rayyann.ai , will be used to detect duplicates and keep track of references.

15. URL to search strategy:

An example search for PubMed yielding 404 results from 1981 to May 1, 2021 is shown below.

(("context$aware*"[Title/Abstract] OR "situation$aware*"[Title/Abstract]) AND ((health*[Title/Abstract] OR medic*[Title/Abstract] OR hospital*[Title/Abstract] OR well*[Title/Abstract] OR diagnos*[Title/Abstract] OR detect*[Title/Abstract] OR clinic*[Title/Abstract] OR condition[Title/Abstract])) AND (patient*[Title/Abstract] OR doctor*[Title/Abstract] OR nurse*[Title/Abstract] OR elder*[Title/Abstract] OR participant[Title/Abstract] OR physician*[Title/Abstract]))

https://pubmed.ncbi.nlm.nih.gov/?term=%28%28%22context%24aware*%22%5BTitle%2FAbstract%5D+OR+%22situation%24aware*%22%5BTitle%2FAbstract%5D%29+AND+%28%28health*%5BTitle%2FAbstract%5D+OR+medic*%5BTitle%2FAbstract%5D+OR+hospital*%5BTitle%2FAbstract%5D+OR+well*%5BTitle%2FAbstract%5D+OR+diagnos*%5BTitle%2FAbstract%5D+OR+detect*%5BTitle%2FAbstract%5D+OR+clinic*%5BTitle%2FAbstract%5D+OR+condition%5BTitle%2FAbstract%5D%29%29+AND+%28patient*%5BTitle%2FAbstract%5D+OR+doctor*%5BTitle%2FAbstract%5D+OR+nurse*%5BTitle%2FAbstract%5D+OR+elder*%5BTitle%2FAbstract%5D+OR+participant%5BTitle%2FAbstract%5D+OR+physician*%5BTitle%2FAbstract%5D%29%29&filter=pubt.journalarticle

16. Condition or domain being studied:

Context aware systems; healthcare; applications used by patients; applications used by healthcare providers; Non lab prototypes

17. Participants/population:

Healthcare providers, caregivers, and patients of any age

18. Intervention(s)/exposure(s):

Context aware technologies, defined as systems where the applications have the "ability to adapt to changing circumstances and respond according to the context of use (J. Kjeldskov, M. Skov, Supporting work activities in healthcare by mobile electronic patient records, in: Proceedings of the 6th Asia–Pacific Conference on Human–Computer Interaction, APCHI 2004, Rotorva, New Zealand, 2004). Context is defined in the usual way, by Dey, as "any information that can be used to characterize the situation of entities (i.e. whether a person, place or object) that are considered relevant to the interaction between a user and an application, including the user and the application themselves." (A. Dey, G. Abowd, D. Salber, A conceptual framework and toolkit for supporting the

rapid prototyping of context-aware applications in special issue on context-aware c) We further define the technology/intervention by focusing only on those applications with a medical focus which have been used by patients to help manage their disease or healthcare providers to help with their workflow or management of patients conditions. Thus, lab prototypes that have not been used by patients/healthcare providers are excluded.

19. Comparator(s)/control(s):

Any comparator.

20. Types of study to be included initially:

All studies featuring primary data will be included. Systematic reviews were eligible and will be evaluated for any missing references but will not be extracted.

21. Context:

Studies with technology field tested in patients within their homes/daily lives or location of treatment, and those used by healthcare providers in their work environment.

22. Primary outcome(s):

Any outcome relevant to improving the quality of life of patients or their management by healthcare providers.

23. Secondary outcome(s):

N/A

24. Data extraction (selection and coding):

Selection – two screeners will independently evaluate eligibility criteria for both title and abstract and for full text citations at each stage of the screening process. Screening software (Rayyan.ai) will be piloted on a randomly-selected subset of references to ensure consistency between screeners prior to using the software to screen all studies returned from the search. Level of agreement (kappa score) will be evaluated to assess level of agreement between screeners.

Extraction – A standardized form will be generated using excel for data abstraction. We will abstract general characteristics of studies such as the sample size, setting, eligibility criteria, participant description, contexts used, technology used, and funding source. Additionally, the TIDieR checklist will be adapted for extracting information about the characteristics of how the context-aware technology was used to assist healthcare providers or patients.[2]

25. Risk of bias (quality) assessment:

Studies will not be assessed for risk of bias as per the Preferred Reporting Items for Systematic Reviews and Meta-Analysis: extension for Scoping Reviews (PRISMA-ScR) guidelines.[3]

26. Strategy for data synthesis:

The findings of this scoping review will be summarized and presented in tables. The resulting papers will be analyzed according to the contexts used, technology present, and type of application the context aware system was used. The purpose of each study's context-aware system wi be described along with the current state of the technology (e.g large field test or early testing by patients/healthcare providers). Areas that could benefit from potential future research and gaps in the current literature will be identified.

27. Analysis of subgroups or subsets:

Context aware applications categorized/analyzed by application type

28. Type of review:

Scoping review

29. Language:

English

30. Country:

All countries

31. Other registration details:

N/A

32. Reference and/or URL for published protocol:

N/A

33. Dissemination plans:

Dissemination of the findings of this review will be in the form of a published manuscript.

34. Keywords:

Context aware; situation aware; healthcare; telemedical systems; patient; healthcare provider

35. Details of any existing review of the same topic by the same authors:

N/A

36. Current review status:

Complete

37. Any additional information:

N/A

38. Details of final report/publication(s):

N/A

References

1. Aromataris E, Munn Z (Editors). *JBI Manual for Evidence Synthesis*. JBI; 2020. Available from https://synthesismanual.jbi.global. doi: 10.46658/JBIMES-20-01Tricco AC,

2. Hoffmann TC, Glasziou PP, Milne R, et al. Better reporting of interventions: template for intervention description and replication (TIDieR) checklist and guide. *BMJ*. 2014;348:g1687. doi: 10.1136/bmj.g1687

3. Lillie E, Zarin W, O'Brien KK, Colquhoun H, Levac D, et al. PRISMA Extension for Scoping Reviews (PRISMAScR): Checklist and Explanation. *Ann Intern Med.* 2018;169:467–473. doi: 10.7326/M18-0850.

1. IEEE Xplore
    a. ("Document Title":context-aware AND (("Abstract":"medic*" OR "Abstract":"health*" OR "hospital") OR ("Abstract":"diagnos*" OR "Abstract":"patient*" OR "detect" OR "doctor" OR "clinic*")))
2. Science Direct
    a. Title, abstract or author-specified keywords: (patient OR diagnose OR detect OR doctor OR clinic) AND (medical OR health OR medicine OR hospital)
    b. Title: Context-aware
3. SpringLink
    a. '(health* OR medic* OR hospital* OR well* OR diagnos* OR detect* OR clinic* OR condition) AND (patient* OR doctor* OR nurse* OR elder* OR participant OR physician*) AND (context NEAR/1 aware OR context NEAR/1 awareness OR situation NEAR/1 aware OR situation NEAR/1 awareness)'
4. Wiley
    a. ""context-aware"" in Title and "(health* OR hospital OR medic*)" in Abstract and "(patient OR diagnos* OR detect OR clinic* OR doctor)" in Abstract
5. EBSCO
    a. TI context-aware AND AB (patient OR diagnos OR detect OR clinic OR doctor) AND AB (health OR medic OR hospital OR healthcare OR medical)
6. ACM Digital Library
    a. [**Publication Title**: context-aware] **AND** [[**Abstract**: health*] **OR** [**Abstract**: medic*] **OR** [**Abstract**: hospital]] **AND** [[**Abstract**: patient*] **OR** [**Abstract**: diagnos*] **OR** [**Abstract**: detect] **OR** [**Abstract**: clinic*] **OR** [**Abstract**: doctor]]
7. PubMed
    a. (("context-aware"[Title]) AND ((patient[Text Word] OR diagnos*[Text Word] OR detect[Text Word] OR clinic*[Text Word] OR doctor[Text Word])) AND (health*[Text Word] OR medic*[Text Word] OR hospital[Text Word]))
